# Health, public sector service use and related costs of Swedish preschool children: results from the *Children and Parents in Focus* trial

**DOI:** 10.1007/s00787-018-1185-1

**Published:** 2018-06-20

**Authors:** Filipa Sampaio, Richard Ssegonja, Camilla Nystrand, Inna Feldman

**Affiliations:** Department of Public Health and Caring Sciences, Uppsala University, BMC, Husargatan 3 (Entry A11), 751 22 Uppsala, Sweden

**Keywords:** Service use, Costs, Mental health, Physical health, Children, Sweden

## Abstract

Despite Sweden’s good child health statistics, data on the mental health and wellbeing of Swedish preschool children is scarce and not routinely collected in healthcare. The study aimed to: identify the proportion of preschool children with mental health and somatic problems, the public sector services used by these children and whether they differ by type of problems, investigate whether other factors affect service use, and estimate the costs associated with these services. This study used cross-sectional data on a sample of 3175 children aged 3–5 from the “Children and Parents in Focus trial”. Data on service use, child health and demographics were obtained from primary caregivers. Child mental health was assessed by both primary caregivers and teachers. 8.9% of the sample reported mental health problems, and approximately 1% had comorbid somatic and mental health problems. Over 50% of the preschoolers used any service, with school assistant being the most frequently used. The average annual cost per child, regardless of health status, was US$921, with 75% of the costs accruing at school. The presence of both somatic and mental health problems predicted higher service use, in particular extra services used at school and at home (mean annual cost US$13826 and US$1583, respectively). Children with comorbid problems accounted for the highest mean costs. Mental health problems among preschool children were particularly high compared to studies from other countries. There is a need to strengthen school mental health services to engage in proactive early identification of children with mental health problems so that appropriate care is provided.

*Trial registration number*: ISRCTN16513449. Registered 23 July 2013.

**Electronic supplementary material:**

The online version of this article (10.1007/s00787-018-1185-1) contains supplementary material, which is available to authorized users.

## Introduction

Children in Sweden are generally healthy [1], as cited by the United Nations Convention of the Rights of the Child: “In an international perspective, the situation among children and young people in Sweden is generally good.” Mental health among children and young people is, however, an exception, with up to 50% of Swedish children aged 11–15 years reporting mental health problems [2]. The mental health among children has, therefore, become a major public health concern, and an increasingly important area for professional and political initiatives. If left untreated, mental health problems can continue into adulthood and are associated with several negative outcomes, such as school dropout, alcohol and drug abuse, poor relationships, and unemployment [3–6]. Mental health problems amount to a large disease [7] and economic burden to the individuals and society, with individuals using more health and other sector resources than their healthy counterparts. For instance, antisocial behaviors are known to result in excessive use of resources in different sectors of society, such as healthcare, educational and justice services [8, 9]. Childhood anxiety disorders are also known to yield large productivity losses of parents due to absence from paid work [10]. Unarguably, early identification of child mental health problems can give place to early intervention, thus improve child health and wellbeing and result in socioeconomic benefits [11]. The recognition of the impacts of mental health problems in the child and adolescent population has prompted the Swedish government to increase investment to finance a multi-sectorial approach for the improvement of child and adolescent mental health [12].

Despite Sweden’s good child health statistics, data on the mental health and wellbeing of Swedish preschool children is scarce [13] and not routinely collected in health care. Research from neighbouring countries show a prevalence of mental health problems among preschool children ranging between 4.8% in Denmark [14] and 7.1% in Norway [15]. Data on costs associated with physical and mental health problems among Swedish preschool children are also lacking. To fill this gap, we have used cross-sectional data from a trial—“Children and Parents in Focus trial” [16] conducted in Uppsala municipality in Sweden. The study is based on all children aged 3–5 years recruited to the study during the period 2013–2017, to provide an overview of the health status and services used by this population of Swedish preschool children.

The aims of this study were to (1) identify the proportion of children with mental health and somatic problems in this population, (2) describe the public sector services used by these children and whether they differ by type of problems, (3) investigate whether other factors affect service use, and (4) estimate the costs associated with these services.

## Methods

### Study design and participants

The “Children and Parents in Focus trial” was conducted during 2013-2017 in Uppsala County. In brief, during the period 2013–2017, parents of children aged 3–5 years were consecutively recruited via Child Health Centres (CHC) in Uppsala County prior to their yearly check-ups. All CHC and administrative heads in Uppsala County were invited to participate in the trial via passive consent. Prior to the child’s yearly check-up, CHC nurses sent out a reminder to the child´s primary caregiver along with two packages of questionnaires. Upon agreement to participate in the study, parents were requested to bring one package to the check-up along with a signed consent form, and to take the other package to the child’s preschool teacher. Preschool teachers were requested to return the completed questionnaire to the CHC nurse in a prepaid envelope. Both primary caregivers and co-parents completed the questionnaires with information concerning parent and child sociodemographic variables, and parenting behaviour and engagement. Primary caregivers provided additional information regarding child and parental health and service use. Preschool teachers completed questionnaires concerning child health.

The current study is a cross-sectional study based on data collected between August 2015 and August 2016. The study population included 3175 children aged 3–5 years old whose parents attended the yearly check-up and agreed to participate in the study, of the 7372 eligible children of the same age in Uppsala County during that period (43%). Data were collected for these children prior to randomisation to any of the arms of the trial, thus outcomes are not influenced by allocation. Further details have been described in detail elsewhere [16].

### Measurements

#### Sociodemographic variables

Background information for both children and parents was collected, including child age and gender, parent age, length of stay in Sweden, ethnicity, marital status, employment, education and living arrangement. Parental information was compared with demographic data from national statistics [17] to understand how representative this sample of parents was of the adult population in Uppsala County.

#### Health outcomes

##### Child mental health status

Mental health status in children was measured using the Strengths and Difficulties Questionnaire (SDQ) [18] by three different responders, mothers, fathers and preschool teachers. The SDQ assesses prosocial, emotional, and behavioural problems in children aged 3–16 years. It consists of 25 items that are subdivided into 5 different scales: emotional symptoms, conduct problems, hyperactivity/inattention, peer relationship problems and prosocial behaviour. A total score can be generated by adding together the scores of the first four sub-scales. The SDQ has demonstrated good internal consistency, reliability [19] and discriminant validity [20], and is deemed appropriate for screening of child behaviour problems [21] by both parents and teachers. Unpublished norms derived from a Swedish sample of 2–5 year olds were used to establish clinical cut-offs [22].

##### Child somatic status

The parents’ questionnaires included a question on whether the child had any chronic illness or an ongoing somatic diagnosis.

##### Parental mental health status

Parental mental health status was assessed using the General Health Questionnaire (GHQ-12) [23], a commonly used self-report measure of psychological distress, which has been validated for use in mental health populations [24]. It comprises 12 items, with higher scores indicating psychological morbidity.

#### Service use

Parents were asked about a range of services used by their children for both somatic and mental health problems in the 12 months prior to the administration of the questionnaire. The services included comprised visits to the general practitioner (GP), inpatient care visits, extra help at school (e.g., special education teacher, counsellor, psychologist, assistant, physiotherapist, other help), and extra help at home (e.g., speech and language therapist, social welfare officer, psychologist, physiotherapist, child welfare officer, contact person, other help).

### Costs

To monetize the services used by children, unit costs for the different types of services were collected from Swedish national statistics [25]. Health care related costs, e.g., GP visits, inpatient care, visits to psychologist and physiotherapist, were valued based on Uppsala County price list of health services [26]. Non-medical resources used in school and at home were based on national average salaries of the corresponding services and overhead rates from Uppsala municipality. To estimate the cost of total services used in school and at home, an average of the included service components was used. A reference year of 2016 was used, and prices were converted from Swedish Krona (SEK) to US dollars (USD) with an exchange rate of 1 USD = 8.82 SEK [27]. Total costs of resources used were estimated through a bottom-up method by multiplying the total number of services used with the 2016 unit costs of the corresponding services.

### Analyses

All base-case analyses were based on parents’ reports, as proxies, of child health status and service use. Only the primary caregiver of the child was included, as this was the only parent for whom full data were available. The dataset was cleaned to exclude possible duplicates. Parents SDQ ratings were used to identify whether children had mental health problems by the application of gender- and age- specific cut-off values based on Swedish norms [28]. As a result, the sample was subdivided into four groups based on the presence of either mental health problems or somatic problems, both mental health and somatic problems or no problems. Firstly, the amount of services used by children by type of provider in the four different groups was examined, and the proportion of children who used services, the total number of visits and the average number of visits per child were reported. Next, three models of analysis were conducted. The first model explored whether mental and somatic problems were predictors of extra help at school and at home by children without controlling for any potential covariates. The second model included the demographic variables, child age and sex as covariates, and the third model included, additionally, parental mental status as a covariate. Linear regression analyses were used when investigating the differences in mean number of visits between each problem group and the reference group (no problems). Logistic regressions were used to investigate whether type of problems were predictors of health and school service use. In the regression analyses, all types of service providers included in the umbrella “extra help at school” and “extra help at home” were grouped together due to the small number of children receiving these services at the service provider level.

Sensitivity analyses were performed using preschool teacher SDQ ratings to investigate whether different informant ratings of child emotional and behavioural problems had an impact on the base-case results. An inter-rater reliability analysis was conducted to investigate the degree of agreement between both informants (parents and preschool teachers). All analyses were undertaken using R studio version 3.2.3.

## Results

### Socioeconomic and demographic data

Characteristics of the sample are shown in Table 1. Out of a total of 3175 surveyed children, full data on parent-rated health status of the children were missing for 85 individuals, resulting in a final sample of (*n* = 3090) children between the ages 3–5. Eighty-five percent of the children did not experience any problems according to parental report, while mental ill-health was reported by 8.9% of the sample. The comparison to teacher ratings showed similar results (84.3 and 8.9%, respectively). The observed agreement statistic between the parents’ and teachers’ ratings was low (30%). Around 1% of the sample was found to have comorbid somatic and mental problems. Fewer girls were represented in the total sample (48.4%), who also experienced less mental and somatic problems than boys, according to both parental and teacher ratings. Most parents in our study sample were born in Sweden and were living together with the other parent. In addition, they had formal employment and had completed more than basic education. In comparison to the population in Uppsala County and in the whole of Sweden, parents in the studied sample were more highly educated, and individuals born outside of Sweden were underrepresented [17].

**Table 1:** Baseline characteristics of surveyed children (3–5 year olds) and their parents

	Boys	Girls	Parents
Child gender (*n*/%)	1637 (51.6)	1536 (48.4)	–
Child age (Mean years/SD)	4.00 (0.81)	4.00 (0.81)	–
Child health status (*n*/%) parent rated			
No problems	1325 (83.4)	1299 (86.5)	–
Mental ill-health only	143 (9.0)	131 (8.7)	–
Somatic ill-health only	92 (5.8)	59 (3.9)	–
Both	28 (1.8)	13 (0.9)	–
Child health status (*n*/%) teacher rated			
No problems	968 (82.5)	655 (87.2)	–
Mental ill-health only	119 (10.1)	54 (7.2)	–
Somatic ill-health only	71 (6.0)	37 (4.9)	–
Both	16 (1.4)	5 (0.7)	–
Parental mental health (*n*/%)			
Probable psychiatric case	29 (1.8)	19 (1.2)	–
No problems	1608 (98.2)	1517 (98.8)	–
Parent age (Mean/SD)			36.63 (5.20)
Duration in Sweden (Mean years/SD)			25.06 (12.84)
Ethnicity (*n*/%)			
Swedish born			2620 (82.81)
Non-Swedish born			457 (14.44)
Marital status (*n*/%)			
Single			186 (5.88)
Other arrangements			2905 (91.81)
Employment (*n*/%)			
Formal income			3087 (97.56)
No formal income			77 (2.43)
Education^a^ (*n*/%)			
< Basic education			7 (0.22)
> Basic education			3014 (95.26)
Living arrangement (*n*/%)			
Both parents			2819 (89.10)
Other arrangements			273 (8.63)

^a^Basic education was defined as having completed at least 9 years of schooling

### Services used by all 3–5 year-olds

#### Amount of services used by type of problem and service provider independent of other factors

Overall, 57% of all 3–5 year-olds used any type of service within or outside of the health care sector during the past 12 months, as shown in Table 2. The specific service with the highest average number of hours used per child was the school assistant. Children with any type of problem visited a GP more than once annually and significantly more than children without problems. Mean number of visits were higher for services provided at school than at home across all health status groups. Among the 274 children with only mental health problems, the total amount of services used at school and at home was not significantly higher than for children without problems. Children who had somatic problems or comorbid mental and somatic problems, on the other hand, used more services both at school and at home. Inpatient care was most frequently used by children experiencing somatic problems.

**Table 2:** Parent-/carer-reported service use in the past 12 months among 3–5 year-olds with/without mental problems and/or somatic problems (*n* = 3090), by type of service use

	Type of problems				All children
	Mental health and somatic problems	Mental health problems only	Somatic problems only	No problems	
Type of service	(*n* = 41)	(*n* = 274)	(*n* = 151)	(*n* = 2624)	(*n* = 3090)
General practitioner (GP)					
*n* (visited the GP)	3	167	128	1370	1700
Total number visits (count)	91	323	391	2532	3337
Average *n* visits per child (SD)	2.22 (2.38)	1.18 (1.47)	2.59 (3.47)	0.96 (1.47)	1.07 (1.62)
Mean difference unadjusted (95% CI)^a^	1.25 (0.77–1.74)***	0.21 (0.02-0.41)*	**1.62 (1.37**–1.88)***	Reference	
Mean difference adjusted^1^^a^	1.23 (0.74–1.72)***	0.21 (0.02-0.41)*	**1.61 (1.36**–1.87)***	Reference	
Mean difference adjusted^2^^a^	1.25 (0.77–1.74)***	0.22 (0.02-0.42)*	**1.61 (1.35**–1.87)***	Reference	
Inpatient care					
*n* (received inpatient care)	4	10	20	53	87
Total number visits (hours)	7	18	141.5	78	244.5
Average n visit hours per child (SD)	0.17 (0.80)	0.07 (0.54)	0.94 (7.45)	0.03 (0.28)	0.08 (1.66)
Mean difference unadjusted (95% CI)^a^	0.14 (− 0.37**–**0.66)	0.04 (− 0.17**–**0.24)	0.91 (0.63–1.18)***	Reference	
Mean difference adjusted^1^^a^	0.13 (− 0.38**–**0.65)	0.04 (− 0.17**–**0.24)	0.90 (0.63–1.18)***	Reference	
Mean difference adjusted^2^^a^	0.14 (− 0-38**–**0.66)	0.04 (− 0.17**–**0.25)	0.90 (0.63–1.18)***	Reference	
Extra help at school					
Special education teacher					
n (visited the special education teacher)	9	9	7	11	36
Total number visits (hours)	819	0	0	429	1248
Average n visits per child (SD)	19.98 (121.82)	0	0	0.16 (8.37)	0.47 (16.20)
Mean difference unadjusted (95% CI)^a^	19.81 (14.91–24.71)***	− 0.16 (− 2.14**–**1.81)	− 0.16 (− 2.77–2.44)	Reference	
Mean difference adjusted^1^^a^	19.73 (14.82–24.63)***	− 0.17 (− 2.15**–**1.80)	− 0.22 (− 2.83–2.39)	Reference	
Mean difference adjusted^2^^a^	19.99 (15.06–24.92)***	− 0.09 (− 2.08**–**1.89)	− 0.23 (− 2.83–2.38)	Reference	
Counsellor^b^					
*n* (visited the counsellor)	0	0	1	0	1
Psychologist^b^					
*n* (visited the psychologist)	0	2	0	3	5
Assistant at school					
*n* (visited the assistant at school)	5	1	6	0	12
Total number visits (hours)	2925	1560	4290	0	8775
Average *n* visits per child (SD)	71.34 (260.73)	5.69 (94.24)	28.41 (190.26)	0	2.76 (58.57)
Mean difference unadjusted (95% CI)^a^	71.34 (53.28–89.41)***	5.69 (− 1.59–12.98)	**28.41 (18.81**–38.02)***	Reference	
Mean difference adjusted^1^^a^	71.08 (53.00–89.16)***	5.66 (− 1.62–12.95)	**28.23 (18.62**–37.84)***	Reference	
Mean difference adjusted^2^^a^	72.22 (54.04–90.39)***	6.00 (– 1.31–13.31)	**28.18 (18.57**–37.79)***	Reference	
Physiotherapist^b^					
*n* (visited the physiotherapist)	1	1	1	0	3
Total number visits (hours)	39	0	0	0	39
Average *n* visits per child (SD)	0.95 (6.09)	0	0	0	0.01 (0.69)
Others					
*n* (visited other professionals)	6	10	18	40	74
Total number visits (hours)	2886	936	5947	825.24	10,594.24
Average n visits per child (SD)	70.39 (307.86)	3.42 (47.32)	39.39 (224.86)	0.31 (3.41)	3.43 (62.56)
Mean difference unadjusted (95% CI)^a^	70.08 (50.82–89.33)***	3.10 (− 4.67–10.87)	**39.07 (28.83**–49.31)***	Reference	
Mean difference adjusted^1^^a^	69.57 (50.29–88.85)***	3.05 (− 4.71–10.82)	38.78 (28.53–49.04)***	Reference	
Mean difference adjusted^2^^a^	70.61 (51.23–89.99)***	3.36 (− 4.43–11.15)	**38.74 (28.49**–48.99)***	Reference	
Total extra help at school					
*n* (total extra help at school)	15	17	23	51	106
Total number visits (hours)	6669	2496	10,237	1254.24	20,656.24
Average *n* visits per child (SD)	162.66 (403.98)	9.11 (105.27)	67.79 (291.40)	0.48 (9.04)	6.68 (87.33)
Mean difference unadjusted (95% CI)^a^	162.18 (135.83–188.53)***	8.63 (− 2.00–19.26)	**67.32 (53.31**–81.33)***	Reference	
Mean difference adjusted^1^^a^	161.33 (134.96–187.70)***	8.55 (− 2.08–19.17)	**66.79 (52.77**–80.82)***	Reference	
Mean difference adjusted^2^^a^	163.77 (137.27–190.27)***	9.27 (− 1.38–19.93)	**66.67 (52.67**–80.71)***	Reference	
Extra help at home					
Speech and language therapist					
*n* (visited the speech and language therapist)	12	14	16	60	102
Total number visits (hours)	13	27	50	209	299
Average n visits per child (SD)	0.32 (1.17)	0.10 (0.65)	0.33 (1.32)	0.08 (0.77)	0.10 (0.80)
Mean difference unadjusted (95% CI)^a^	**0.24 (**− 0.01–0.49)***	0.02 (− 0.08–0.12)	0.25 (0.12–0.38)	Reference	
Mean difference adjusted^1^^a^	**0.23 (**− 0.02–0.47)***	0.02 (− 0.08–0.12)	0.24 (0.11–0.38)	Reference	
Mean difference adjusted^2^^a^	**0.24 (**− 0.01–0.49)***	0.02 (− 0.08–0.12)	0.24 (0.11–0.38)	Reference	
Counsellor^b^					
*n* (visited the counsellor)	1	0	1	0	2
Total number visits (hours)	0	0	4	0	4
Average *n* visits per child (SD)	–	–	0.03 (0.33)	–	0.001 (0.07)
Psychologist					
*n* (visited the psychologist)	7	8	1	7	23
Total number visits (hours)	13	10	0	17	40
Average n visits per child (SD)	0.32 (0.93)	0.04 (0.28)	0	0.01 (0.17)	0.01 (0.21)
Mean difference unadjusted (95% CI)^a^	0.31 (0.25**–**0.37)	0.03 (0.00-0.06)***	**–** 0.01 (**–** 0.04–0.03)	Reference	
Mean difference adjusted^1^^a^	0.31 (0.25–0.37)***	0.03 (0.00-0.06)***	**–** 0.01 (**–** 0.04–0.03)	Reference	
Mean difference adjusted^2^^a^	0.32 (0.25–0.38)***	0.04 (0.01-0.06)***	**–** 0.01 (**–** 0.04–0.03)	Reference	
Physiotherapist					
*n* (visited the physiotherapist	7	3	4	6	20
Total number visits (hours)	8	4	15	8	35
Average *n* visits per child (SD)	0.20 (0.56)	0.01 (0.17)	0.10 (0.65)	0.003 (0.08)	0.01 (0.25)
Mean difference unadjusted (95% CI)^a^	0.19 (0.14–0.25)***	0.01 (− 0.01**–**0.04)	0.10 (0.07–0.13)***	Reference	
Mean difference adjusted^1^^a^	0.19 (0.13–0.25)***	0.01 (− 0.01**–**0.03)	0.09 (0.07–0.12)***	Reference	
Mean difference adjusted^2^^a^	0.19 (0.14–0.25)***	0.01 (− 0.01**–**0.03)	0.09 (0.07–0.12)***	Reference	
Child welfare officer					
*n* (visited the child welfare officer)	2	3	0	1	6
Total number visits (hours)	0	1	0	6	7
Average *n* visits per child (SD)	0	0.004 (0.06)	0	0.002 (0.12)	0.002 (0.11)
Mean difference unadjusted (95% CI)^a^	− 0.002 (− 0.36**–**0.03)	0.001 (− 0.01**–**0.01)	**–** 0.002 (**–** 0.02**–**0.02)	Reference	
Mean difference adjusted^1^^a^	− 0.001 (− 0.04**–**0.03)	0.001 (− 0.02**–**0.02)	**–** 0.002 (**–** 0.02**–**0.02)	Reference	
Mean difference adjusted^2^^a^	− 0.001 (− 0.04**–**0.03)	0.002 (− 0.01**–**0.02)	**–** 0.002 (**–** 0.02**–**0.02)	Reference	
Contact person (voluntary services)^b^					
*n* (visited the contact person)	1	1	0	0	2
Others					
*n* (visited other professionals)	6	2	7	11	26
Total number visits (hours)	320.75	0	375	69	764.75
Average *n* visits per child (SD)	7.82 (48.71)	0	2.48 (29.70)	0.03 (0.80)	0.24 (8.55)
Mean difference unadjusted (95% CI)^a^	7.80 (5.14–10.45)***	− 0.03 (− 1.10**–**1.05)	2.46 (1.04–3.87)***	Reference	
Mean difference adjusted^1^^a^	7.77 (5.11–10.43)***	− 0.03 (− 1.10**–**1.04)	2.44 (1.02–3.85)***	Reference	
Mean difference adjusted^2^^a^	7.88 (5.20–10.55)***	0.00 (− 1.07**–**1.08)	2.43 (1.02–3.85)***	Reference	
Total extra help at home					
*n* (extra help at home)	16	22	17	77	181
Total number visits (hours)	354.75	42	444	309	1149.75
Average *n* visits per child (SD)	8.65 (48.65)	0.15 (0.82)	2.94 (29.73)	0.12 (1.24)	0.37 (8.62)
Mean difference unadjusted (95% CI)^a^	8.53 (5.86–11.21)***	0.04 (− 1.04**–**1.11)	2.82 (1.40–4.24)***	Reference	
Mean difference adjusted^1^^a^	8.49 (5.82–11.17)***	0.03 (− 1.05**–**1.11)	2.79 (1.37–4.22)***	Reference	
Mean difference adjusted^2^^a^	8.62 (5.94–11-31)***	0.07 (− 1.01**–**1.15)	2.79 (1.37–4.21)***	Reference	
Any service					
Total number of children (count)	38	177	132	1417	1764
Average *n* visits per child (SD)	0.93 (0.26)	0.69 (0.46)	0.90 (0.30)	0.59 (0.49)	0.62 (0.49)
Mean difference unadjusted (95% CI)^a^	0.34 (0.19–0.49)***	0.10 (0.04–0.16)**	0.31 (0.23–0.38)***	Reference	
Mean difference adjusted^1^^a^	0.33 (0.18–0.48)***	0.10 (0.04–0.16)**	0.31 (0.23–0.39)***	Reference	
Mean difference adjusted^2^^a^	0.32 (0.17–0.47)***	0.10 (0.04–0.16)**	0.31 (0.23–0.39)***	Reference	

*SD* standard deviation, *CI* confidence interval

^1^Adjusted for age and gender (95% CI)

^2^Adjusted for age, gender and parental mental health (95% CI)

^a^Mean difference in mean number of visits from linear regression analysis

^b^Mean differences not estimated due to null usage

*p < 0.05, **p < 0.01, ***p < 0.001

#### Likelihood of using services by type of problems and service provider independent of other factors

The likelihood of service use based on children’s mental and somatic health is presented in Table 3, where type of service has been divided into four categories due to the small sample sizes of each individual service type: use of a general practitioner, use of inpatient care, services used at school and at home. Univariate regression analyses showed significant relationships between health status of children and service use, seen in the first part of Table 3. In particular, children who had both mental and somatic problems had significantly higher odds of using any type of service compared to children without problems [odds ratio (OR) = 8.97, 95% confidence interval (CI)  (3.24, 37.23)]. Relative to having no problems, children with mental health problems only were significantly more likely to use any type of services, although at small odds (OR = 1.55, 95% CI (1.18, 2.05)]. Looking at each of the type of services used separately, a majority of them were significantly more likely to be used by children with any problems.

**Table 3:** Mental and physical health of children and mental health of parents as predictors of health and school service use in the past 12 months among 3–5 year-olds (*n* = 3090)

	Type of Problems			
	Mental health and somatic problems	Mental health problems only	Somatic problems only	No problems
	(*n* = 41)	(*n* = 274)	(*n* = 151)	(*n* = 2624)
Type of service	OR(CI)	OR(CI)	OR(CI)	
Model 1				
General practitioner	6.10 (2.61–17.81)***	1.41 (1.10–1.83)**	4.85 (3.15–7.79)***	Reference group
Inpatient care	5.32 (1.55–13.90)**	1.84 (0.87**–**3.51)	7.37 (4.19–12.51)***	Reference group
Extra help at school	31.62 (15.21–64.62)***	3.38 (1.87–5.83)***	8.70 (5.06–14.58)***	Reference group
Extra help at home	21.93 (11.02–42.67)***	2.89 (1.73–4.65)***	4.17 (2.33–7.09)***	Reference group
Any services	8.97 (3.24–37.229)***	1.55 (1.18–2.05)**	6.24 (3.76–11.15)***	Reference group
Model 2				
General practitioner	5.93 (2.53-17.35)***	1.41 (1.09–1.83)**	4.80 (3.11–7.73)***	Reference group
Inpatient care	5.14 (1.49–13.46)**	1.84 (0.87**–**3.50)	7.22 (4.10–12.27)***	Reference group
Extra help at school	28.72 (13.67–59.34)***	3.31 (1.82–5.72)***	8.25 (4.78–13.88)***	Reference group
Extra help at home	22.40 (10.97–44.97)***	2.98 (1.77–4.82)***	4.06 (2.24–6.98)***	Reference group
Any services	8.66 (3.12–35.95)***	1.55 (1.18–2.05)**	6.12 (3.68-10.95)***	Reference group
Model 3				
General practitioner	5.92 (2.51–17.37)***	1.41 (1.09–1.83)*	4.80 (3.12–7.73)***	Reference group
Inpatient care	5.01 (1.43–13.37)**	1.82 (0.86**–**3.49)	7.23 (4.10–12.29)***	Reference group
Extra help at school	26.30 (12.36–54.85)***	3.15 (1.73-5.47)***	8.51 (4.93–14.34)***	Reference group
Extra help at home	24.36 (11.77–49.79)***	3.05 (1.81–4.94)***	4.05 (2.24–6.97)***	Reference group
Any services	8.41 (3.02–34.96)***	1.54 (1.17–2.03)**	6.13 (3.69–10.96)***	Reference group

*OR* odds ratio, *CI* confidence interval

*p < 0.05, **p < 0.01, ***p < 0.001

Model 1—logistic regression, Model 2—logistic regression controlling for child age and gender, Model 3—logistic regression controlling for child age, gender and parental mental health

#### Likelihood of using services by type of problems and service provider by age, gender and parental mental health status

The second and third part of Table 3 incorporate child age, gender and parental mental health as predictors of child service use. In comparison to the first analysis that did not control for these confounders, the results show similar patterns, regarding both direction and magnitude. Children with comorbid somatic and mental health problems were more likely to use services at school compared to healthy children, when controlling for child age and gender [OR = 28.72, 95% CI (13.67, 59.34)], and when additionally controlling for parental mental health [OR = 26.3, 95% CI (12.36, 54.85)].

In summary, all types of services were significantly more likely to be used by children with somatic problems only and comorbid problems (both somatic and mental health problems) compared to the group of children with no problems.

### Costs

All unit costs and corresponding sources are presented in Table 4. Table 5 presents the mean annual cost incurred by children, based on health status. The costs represent services used within the health care setting, at school and at home. The average annual cost per child, regardless of health status, was US$921. The largest cost accrued was related to services received at preschool, representing 60% of the total annual costs. The mean annual costs of visits to the GP and inpatient care were highest among children with somatic problems only (US$515 and US$972, respectively), while having both somatic and mental health problems led to the largest cost burden for services at school and at home (US$13 826 and US$1583, respectively). A majority of the mean annual costs of service use for children aged 3–5 could be accredited to children with comorbid problems. On the other hand, children characterized as having no problems had the lowest annual cost.

**Table 4:** Unit costs (USD 2016 prices)

Resource	Unit cost (USD, 2016)	Source
General practitioner (visit)	199	Price list 2016, Akademiska Children’s Hospital, Uppsala County Council
Inpatient care (day)	1 034	Price list 2016, Akademiska Children’s Hospital, Uppsala County Council
Special education teacher	42	Average hourly wage (2016), employees within Uppsala County municipal sector
Counsellor (at health clinic)	248	Price list 2016, Akademiska Children’s Hospital, Uppsala County Council (45 min visit)
Counsellor (at preschool)	46	Average hourly wage (2016), employees within Uppsala County municipal sector
Psychologist (at health clinic)	299	Price list 2016, Akademiska Children’s Hospital, Uppsala County Council (60 min visit)
Psychologist (at preschool)	55	Average hourly wage (2016), employees within Uppsala County municipal sector
Assistant at preschool	32	Average hourly wage (2016), employees within Uppsala County municipal sector
Physiotherapist	248	Price list 2016, Akademiska Children’s Hospital, Uppsala County Council (45 min visit)
Speech and language therapist	248	Price list 2016, Akademiska Children’s Hospital, Uppsala County Council (45 min visit)
Child welfare officer	46	Average hourly wage (2016), employees within Uppsala County municipal sector
Contact person	11	25% of the average salary in Sweden (Statistics Sweden) + social fees
Average cost of extra help at preschool^a^	85	
Average cost of extra help at home^b^	183	

^a^Calculated as the average unit cost of the services included in school services [special education teacher, counsellor (at preschool), psychologist (at preschool), assistant at preschool and physiotherapist]. Based on salaries, employer social fees and overheads

^b^Calculated as the average unit cost of the services included in services used at home [speech and language therapist, child welfare officer, counsellor (at health clinic), psychologist (at health clinic), physiotherapist and contact person]. Based on salaries, employer social fees and overheads

**Table 5:** Mean (SD) annual cost (USD 2016) of services used by children ages 3–5, by mental and somatic health status

	Type of Problems				All children
	Mental health and somatic problems	Mental health problems only	Somatic problems only	No problems	
	(*n* = 41)	(*n* = 274)	(*n* = 151)	(*n* = 2624)	(*n* = 3090)
Type of service	Mean (SD)	Mean (SD)	Mean (SD)	Mean (SD)	Mean (SD)
General Practitioner					
Based on average number of visits per child	442 (473)	235 (292)	515 (690)	191 (292)	213 (322)
Inpatient care					
Based on the average number of days per child	176 (827)	72 (558)	972 (7701)	31 (289)	83 (1716)
Extra help at school (average per child)					
Special educational teacher	839 (5116)	–	–	7 (352)	20 (680)
Counsellor	–	–	–	–	–
Psychologist	–	–	–	–	–
Assistant	2283 (8343)	182 (3016)	909 (6088)	–	88 (1874)
Physiotherapist	9186 (58,813)	–	–	–	2 (171)
Other	5983 (26,168)	291 (4022)	3348 (19,113)	26 (290)	292 (5318)
Total cost extra help at school	13,826 (34,338)	774 (8948)	5762 (24,769)	41 (768)	568 (7432)
Extra help at home (average per child)					
Speech and language therapist	79 (290)	25 (161)	82 (327)	20 (191)	25 (198)
Counsellor	–	–	7 (82)	–	0.25 (17)
Psychologist	96 (278)	12 (84)	–	3 (51)	3 (63)
Physiotherapist	50 (139)	2 (42)	25 (161)	0.74 (20)	2 (62)
Child welfare officer	–	0.18 (2)	–	0.09 (5)	0.092 (5)
Contact person	–	–	–	–	–
Other	1431 (8914)	–	446 (5351)	5 (14)	43 (1538)
Total cost extra help at home	1583 (8903)	27 (150)	538 (5441)	22 (227)	68 (1577)
Average total cost per child	16,027 (38,768)	1105 (8976)	7785 (28,631)	285 (915)	921 (8422)

*SD* standard deviation

The analyses of service use and costs based on the teachers’ SDQ ratings showed similar results to those based on parents' ratings, as shown in tables A1–3 in the supplementary appendix.

## Discussion

This study aimed at describing the health status, public sector service use and related costs of Swedish preschool children drawing on cross-sectional data from a trial conducted in Uppsala County, Sweden. Of all 3–5 year olds, approximately 8.9% had mental health problems based on both parent and teacher ratings. Despite similar results, this estimate may not be fully representative of the mental health status of these children, as the agreement statistic between the parents’ and teachers’ ratings was low (30%). Parent and teacher ratings are often a reliable and efficient manner of getting a picture of child health and wellbeing across different environments [29]. The literature shows, however, diverging results on agreement between parent and teacher ratings of child health [29], which nevertheless highlights the difficult task of assessing the mental health of such small children and the importance of collecting information from both informants. Studies on the prevalence of mental health problems among preschool children conducted in other Nordic countries show slightly lower prevalence, ranging between 4.8% in a Danish sample [14] to 7.1% in a Norwegian sample [15]. Another study from Germany [30] reported a prevalence of 7.4%.

The prevalence of mental health problems reported by parents in this sample was unexpectedly low (less than 2%) compared to available literature. A nationally representative survey using the GHQ estimated a prevalence of mental health problems of 19% amongst adults aged 35–49 years in Uppsala County [31]. Another recently published study, also using the GHQ, reported mental health problems of 28% amongst parents of young children in Sweden [32]. Reasons for the low rate of mental health problems among the parents in our sample could be various. First, the sample was overrepresented by highly educated individuals, which is known to positively affect mental health [33]. Individuals born outside of Sweden were also underrepresented. A systematic review of Swedish studies looking at the relationship between mental health and immigrant status found that not being born in Sweden was associated with higher odds of depression and/or anxiety [34]. Second, our study may have attracted parents of certain characteristics, who may not be representative of the general population, and who may have self-selected into the study. Importantly, this may strongly suggest that parents with mental health problems may not have access to this type of service, or may experience barriers to participation. This highlights the need to further investigate this issue, so that those who are most in need receive appropriate services.

Over half of the sample of preschoolers used any service within or outside the health care sector, with the most frequently used service being the school assistant. The same was true for the cost burden, with 75% accruing to the school sector. This finding emphasizes the need to strengthen school mental health services to engage in proactive case finding practices to identify students with mental health problems early enough so that appropriate care, e.g., preventive interventions or referral for further care can be done. Mental health problems increase the risk of repeating a grade, truancy, and dropping out of school [35]. Implementing evidence-based preventive programs in schools could contribute to lower school absenteeism, better educational outcomes, and eventual cost reductions [35]. A Swedish study [36] has estimated the yearly cost of additional support at school to a child with mental health problems to amount to approximately 19,000 USD per student, in 2016 prices. This estimate is, unsurprisingly, much higher than the one in our study, given the children in Wellander’s study were older (6–16 years), with identified problems such as ADHD, depression or anxiety, and used more services.

Children with mental health problems only consumed more services at school and at home than children without problems, but this difference was not statistically significant. Notably, children with both somatic and mental health problems significantly used more services at school and at home than children experiencing no problems, contributing to the largest cost burden of this group of children. These results point to somatic problems being the driver of the differences in resource use between the groups. It is important to pinpoint that it may be difficult to detect mental health problems in such young children, and sometimes problems may go unnoticed or regarded as normal for the stage of development of the child. It is, thus, important that different professionals, including psychologists, speech and language therapists, physiotherapists and physicians assess these children so that proper services can be offered.

Inpatient care and visits to the GP were highest among children with somatic problems, and so were the related costs. When investigating predictors of service use, children with any problems were more likely to use any type of services, although the presence of both somatic and mental health problems predicted higher service use, in particular services used at school and at home. These results remained stable even when controlling for child background variables and parental mental health. This suggests that the children needing health care are in fact accessing services. This calls for the need to assess if there are facilities in place to identify these children early and fully manage them.

### Limitations

This study has some limitations that should be taken into account. Despite its fairly large sample size (*n* = 3090) and use of validated instruments, this study had a response rate of about 43%, which may have not given a complete representative picture of the current health status of preschool children in Uppsala County. Thus, results should be interpreted with that in mind. Second, the sample of parents used in this study was not representative of Uppsala County, with a larger proportion of highly educated individuals born in Sweden. Third, the agreement statistics between parent and teacher ratings was low; hence the prevalence of children with mental health problems reported in this study could have been under or overestimated. Fourth, only the primary caregiver of the child was included in this study, as this was the only parent for whom full health and resource use data was available. Ratings from both parents would be important to provide a full picture of the child’s mental health and enhance the sensitivity of identifying children requiring support [37, 38]. Fifth, often children present with symptomatology that overlaps different mental health conditions, but in many cases only one set of symptoms corresponds to defined criteria for a DSM diagnosis to be set. Thus, some conditions may go undiagnosed, which may result in services not being offered, hence underestimate the quantity of service use in our study. Finally, the cross-sectional design of this study does not allow for a comparative analysis over time, thus limiting the usefulness of the results.

### Implications to policy and practice

This study addresses the need for population-based data on the mental health and wellbeing of children of preschool age [13]. In Sweden, it is part of the protocol for nurses to informally ask parents about the mental health of their child upon the early check-up visits to the CHC. A few of the CHC who participated in the “Children and Parents in Focus” trial are currently implementing into praxis the routine of sending out a short version of the SDQ questionnaire to parents together with the reminder to their yearly check-up visits. Allowing parents to fill in the questionnaire online would also be an efficient way to facilitate communication between parents and health care professionals. No unified standardized method or tool is, however, used to assess child mental health at these visits. Having such a tool, would allow data to be registered in a standardized way, and importantly, would allow early identification of child mental health problems. A recent study by Fält et al. [39] has explored nurses', parents' and teachers' perspectives on information sharing of child health using SDQ in Sweden, and has demonstrated that all parties shared a desire to have a complete picture of the child’s health and acknowledged the importance of information sharing. Early identification is important so that the appropriate care can be given to the children who need it, thus contribute to child health and wellbeing. Further, early intervention can help prevent problems from persisting into the future, as well as becoming more severe. Prevention and early intervention are effective in improving child health and wellbeing [11]. Better child health and wellbeing also means greater economic benefits to the children, their families and society due to decreased use of societal resources, and consequently lower costs [11]. Childhood offers the greatest opportunities for positive human development, but is also the period when children are most at risk. Investments to improve child health in early childhood are proven to be one of the best investments a society can make, since intervention costs are returned many times over the lifetime of the child [40]. A Swedish study by Wellander et al. [36], showed that by implementing evidence-based school programs aimed to improve children’s mental health, the municipality as a payer would receive a return on their invested resources in less than 2 years after implementation [36].

Positive interventions in early childhood work best when they bring together a variety of sectors, including health, education, and support for parents. The results highlight the important role of schools in providing mental health resources to these children. Schools are an important arena for service provision and for identification of child mental health problems. Strengthening mental health services in schools could be an additional way towards improving child health and wellbeing. For instance, employing routine screening for common illnesses and mental health concerns by a GP before school start, having a school psychiatric and public health nurse available, and advocating for school-based mental health preventive interventions as a routine activity, in view of the high prevalence of mental health problems in Sweden, are a few examples of how school mental health services could be strengthened.

## Conclusions

Rates of mental health problems among children in preschool age are particularly high compared to studies from other countries. The findings demonstrated that children with both somatic and mental health problems use services mostly at school and at home. This highlights the role of schools as an important arena for service provision and for identification of child mental health problems. There is a need to strengthen school mental health services to engage in proactive early identification of children with mental health problems so that appropriate care is provided.

## Electronic supplementary material

Below is the link to the electronic supplementary material.

Supplementary material 1 (DOCX 31 kb)
