# Supplementary material for: Health, public sector service use and related costs of Swedish preschool children: results from the Children and Parents in Focus trial
Source: Eur Child Adolesc Psychiatry. 2018 Jun 20;28(1):43–56. doi: 10.1007/s00787-018-1185-1 (PMC6349965; doi:10.1007/s00787-018-1185-1)
Supplement: Supplementary file 1 — Supplementary material 1 (DOCX 31 kb) [file 787_2018_1185_MOESM1_ESM.docx]

**Supplementary appendix**

*Results based on teachers’ SDQ ratings*

**Table A1. Table 2.** Parent-/carer-reported service use in past 12 months among 3- to 5-year-olds with/without mental problems and/or somatic problems (n = 3,090), by type of service use

|  | Type of problems | | | | All children |
| --- | --- | --- | --- | --- | --- |
|  | Mental health and somatic problems | Mental health problems only | Somatic problems only | No problems |  |
|  | (n= 21) | (n= 173) | (n= 108) | (n= 1623) | (n= 1925) |
| Type of service |  |  |  |  |  |
| **General Practitioner** |  |  |  |  |  |
| n (visited the GP) | 18 | 89 | 92 | 906 | 1105 |
| Total number visits (count) | 80 | 205 | 241 | 1705 | 2231 |
| Average n visits per child (SD) | 3.81 (4.20) | 1.18 (2.11) | 2.23 (2.42) | 1.05 (1.39) | 1.16 (1.62) |
| Mean difference unadjusted (95% CI)^a^ | **2.76 (2.07-3.45)** | 0.13 (-0.12-0.38) | **1.18 (0.87-1.49)** | Reference |  |
| Mean difference adjusted* ^a^ | **2.75 (2.07-3.44)** | 0.14 (-0.11-0.39) | **1.18 (0.87-1.49)** | Reference |  |
| Mean difference adjusted** ^a^ | **2.76 (2.08-3.45)** | 0.15 (-0.10-0.40) | **1.18 (0.87-1.49)** | Reference |  |
| **Inpatient care** |  |  |  |  |  |
| n (received inpatient care) | 3 | 5 | 10 | 28 | 46 |
| Total number visits (Hours) | 12 | 5 | 31 | 41 | 89 |
| Average n visit hours per child (SD) | 0.57 (2.18) | 0.03 (0.17) | 0.29 (1.41) | 0.03 (0.24) | 0.05 (1.66) |
| Mean difference unadjusted (95% CI) ^a^ | **0.55 (0.35-0.74)** | 0.004 (-0.07-0.08) | **0.26 (0.17-0.35)** | Reference |  |
| Mean difference adjusted* ^a^ | **0.54 (0.34-0.74)** | 0.001 (-0.07-0.07) | **0.26 (0.17-0.35)** | Reference |  |
| Mean difference adjusted** ^a^ | **0.54 (0.34-0.74)** | 0.002 (-0.07-0.07) | **0.26 (0.17-0.35)** | Reference |  |
| **Extra help at school** |  |  |  |  |  |
| ***Special education teacher*** | 4 | 7 | 9 | 7 | 27 |
| n (visited the special education teacher) | 1 | 0 | 1 | 1 | 3 |
| Total number visits (Hours) | 1.86 (8.51) | 0 | 7.22 (75.06) | 0.26 (10.65) | 0.65 (16.20) |
| Average n visits per child (SD) | 1.59 (-7.13-10.32) | -0.26 (-3.44-2.91) | **6.96 (3.01-10.91)** | Reference |  |
| Mean difference unadjusted (95% CI) ^a^ | 1.48 (-7.26-10.21) | -0.35 (-3.53-2.839 | **6.90 (2.95-10.85)** | Reference |  |
| Mean difference adjusted* ^a^ | 1.50 (-7.24-10.24) | -0.33 (-3.52-2.87) | 6.90 (2.95-10.85) | Reference |  |
| Mean difference adjusted** ^a^ |  |  |  |  |  |
| ***Counsellor ^b^*** | 0 | 0 | 1 | 0 | 1 |
| n (visited the counsellor) | 0 | 0 | 0 | 0 |  |
| ***Psychologist ^b^*** | 0 | 1 | 0 | 2 | 3 |
| n (visited the psychologist) | 0 | 0 | 0 | 0 |  |
| ***Assistant at school*** | 3 | 0 | 6 | 0 | 9 |
| n (visited the assistant at school) | 2 | 0 | 4 | 0 | 6 |
| Total number visits (Hours) | 130.0 (415.19) | 0 | 36.11 (197.98) | 0 | 3.44 (58.57) |
| Average n visits per child (SD) | **130 (102.83-157.179** | 0 | **36.11 (23.82)** | Reference |  |
| Mean difference unadjusted (95% CI) ^a^ | **129.44 (102.26-156.62)** | -0.37 (-10.28-9.53) | **35.86 (23.57-48.15)** | Reference |  |
| Mean difference adjusted* ^a^ | **129.67 (102.47-156.86)** | -0.15 (-10.09-9.78) | **35.84 (23.55-48.14)** | Reference |  |
| Mean difference adjusted** ^a^ |  |  |  |  |  |
| ***Physiotherapist*** | 1 | 0 | 1 | 1 | 3 |
| n (visited the physiotherapist) | 1 | 0 | 0 | 0 | 1 |
| Total number visits (Hours) | 1.86 (8.51) | 0 | 0 | 0 | 0.02 (0.69) |
| Average n visits per child (SD) | _ | _ | _ | _ |  |
| ***Others*** | 4 | 11 | 17 | 25 | 57 |
| n (visited other professionals) | 1 | 4 | 12 | 15 | 32 |
| Total number visits (Hours) | 3.71 (17.02) | 5.00 (59.39) | 69.51 (304.37) | 0.35 (3.64) | 4.68 (62.56) |
| Average n visits per child (SD) | 3.37 (-28.55-35.28) | 4.65 (-6.97-16.27) | **69.17 (54.72-83.61)** | Reference |  |
| Mean difference unadjusted (95% CI) ^a^ | 2.88 (-29.06-34.83) | 4.39 (-7.25-16.04) | **69.00 (54.54-83.45)** | Reference |  |
| Mean difference adjusted* ^a^ | 3.02 (-28.96-34.99) | 4.52 (-7.17-16.20) | **68.99 (54.53-83.44)** | Reference |  |
| Mean difference adjusted** ^a^ |  |  |  |  |  |
| **Total extra help at school** | 9 | 16 | 22 | 33 | 80 |
| n (total extra help at school) | 4 | 4 | 15 | 16 | 39 |
| Total number visits (Hours) | 137.43 (413.40) | 5.00 (59.39) | 112.85 (363.51) | 0.61 (11.25) | 8.80 (87.33) |
| Average n visits per child (SD) | **136.82 (94.70-178.94)** | 4.38 (-10.96-19.72) | **112.23 (93.18-131.29)** | Reference |  |
| Mean difference unadjusted (95% CI)a | **135.66 (93.52-177.79)** | 3.67 (-11.68-19.03) | **111.76 (92.70-130.81)** | Reference |  |
| Mean difference adjusted* ^a^ | **136.04 (93.88-178.20)** | 4.04 (-11.39-19.44) | **111.73 (92.67-130.79)** | Reference |  |
| Mean difference adjusted** ^a^ |  |  |  |  |  |
| **Extra help at home** |  |  |  |  |  |
| ***Speech and language therapist*** | 4 | 9 | 17 | 41 | 71 |
| n (visited the speech and language therapist) | 1 | 4 | 10 | 33 | 48 |
| Total number visits (Hours) | 0.24 (1.09) | 0.10 (0.67) | 0.33 (1.16) | 0.08 (0.69) | 0.10 (0.80) |
| Average n visits per child (SD) | 0.16 (-0.16-0.47) | 0.02 (-0.10-0.13) | **0.25 (0.11-0.39)** | Reference |  |
| Mean difference unadjusted (95% CI) ^a^ | 0.15 (-0.16-0.46) | 0.01 (-0.11-0.12) | **0.25 (0.10-0.39)** | Reference |  |
| Mean difference adjusted* ^a^ | 0.15 (-0.16-0.47) | 0.01 (-0.10-0.12) | **0.25 (0.10-0.39)** | Reference |  |
| Mean difference adjusted** ^a^ |  |  |  |  |  |
| ***Counsellor ^b^*** | 0 | 0 | 1 | 0 | 1 |
| n (visited the counsellor) | 0 | 0 | 1 | 0 | 1 |
| Total number visits (Hours) | _ | _ | 0.04 (0.38) | _ | 0.002 (0.07) |
| Average n visits per child (SD) | **_** | **_** | _ | _ |  |
| ***Psychologist*** | 3 | 3 | 3 | 4 | 13 |
| n (visited the psychologist) | 1 | 0 | 3 | 4 | 8 |
| Total number visits (Hours) | 0.19 (0.87) | 0 | 0.06 (0.42) | 0.005 (0.10) | 0.01 (0.21) |
| Average n visits per child (SD) | **0.19 (0.12-0.25)** | **-0.005 (-0.03-0.02)** | 0.06 (0.03-0.09) | Reference |  |
| Mean difference unadjusted (95% CI) ^a^ | **0.18 (0.11-0.25)** | **-0.01 (-0.03-0.02)** | 0.06 (0.03-0.09) | Reference |  |
| Mean difference adjusted* ^a^ | **0.18 (0.11-0.25)** | **-0.01 (-0.03-0.02)** | 0.06 (0.03-0.09) | Reference |  |
| Mean difference adjusted** ^a^ |  |  |  |  |  |
| ***Physiotherapist*** | 2 | 2 | 6 | 6 | 16 |
| n (visited the physiotherapist | 2 | 1 | 4 | 5 | 12 |
| Total number visits (Hours) | 0.24 (0.77) | 0.02 (0.23) | 0.08 (0.48) | 0.005 (0.09) | 0.01 (0.25) |
| Average n visits per child (SD) | **0.23 (0.16-0.31)** | 0.01 (-0.02-0.04) | **0.08 (0.04-0.11)** | Reference |  |
| Mean difference unadjusted (95% CI) ^a^ | **0.23 (0.16-0.31)** | 0.01 (-0.02-0.04) | **0.08 (0.04-0.11)** | Reference |  |
| Mean difference adjusted* ^a^ | **0.23 (0.16-0.31)** | 0.01 (-0.02-0.04) | **0.08 (0.04-0.11)** | Reference |  |
| Mean difference adjusted** ^a^ |  |  |  |  |  |
| ***Child welfare officer*** | 1 | 1 | 1 | 1 | 4 |
| n (visited the child welfare officer) | 0 | 0 | 0 | 1 | 1 |
| Total number visits (Hours) | 0 | 0 | 0 | 0.001 (0.02) | 0.001 (0.11) |
| Average n visits per child (SD) | _ | _ | _ | Reference |  |
| ***Contact person (voluntary services) ^b^*** |  |  |  |  |  |
| n (visited the contact person) | 1 | 0 | 0 | 0 | 1 |
| ***Others*** |  |  |  |  |  |
| n (visited other professionals) | 1 | 3 | 7 | 9 | 20 |
| Total number visits (Hours) | 312 | 0 | 373.75 | 69 | 754.75 |
| Average n visits per child (SD) | 14.86 (68.08)) | 0 | 3.46 (35.12) | 0.04 (1.01) | 0.39 (8.55) |
| Mean difference unadjusted (95% CI) ^a^ | **14.81 (10.14-19.49)** | -0.04 (-1.75-1.66) | **3.42 (1.30-5.53)** | Reference |  |
| Mean difference adjusted* ^a^ | **14.80 (10.12-19.48)** | -0.07 (-1.77-1.64) | **3.40 (1.28-5.52)** | Reference |  |
| Mean difference adjusted** ^a^ | **14.82 (10.14-19.51)** | -0.04 (-1.75-1.67) | **3.40 (1.28-5.51)** | Reference |  |
| **Total extra help at home** |  |  |  |  |  |
| n (extra help at home) | 5 | 12 | 20 | 55 | 92 |
| Total number visits (Hours) | 326 | 20 | 429.75 | 218 | 993.75 |
| Average n visits per child (SD) | 15.52 (67.98) | 0.12 (0.71) | 3.98 (35.129 | 0.13 (1.40) | 0.52 (8.62) |
| Mean difference unadjusted (95% CI) ^a^ | **15.38 (10.70-20.08)** | -0.02 (-1.73-1.69) | **3.84 (1.72-5.97)** | Reference |  |
| Mean difference adjusted* ^a^ | **15.36 (10.67-20.05)** | -0.06 (-1.77-1.65) | **3.82 (1.70-5.94)** | Reference |  |
| Mean difference adjusted** ^a^ | **15.39 (10.70-20.08)** | -0.03 (-1.74-1.69) | **3.82 (1.69-5.94)** | Reference |  |
| **Any service** |  |  |  |  |  |
| Total number of children (count) | 18 | 100 | 96 | 935 | 1149 |
| Average n visits per child (SD) | 0.86 (0.34) | 0.63 (0.48) | 0.91 (0.29) | 0.62 (0.49) | 0.62 (0.49) |
| Mean difference unadjusted (95% CI) ^a^ | **0.24 (0.03-0.44)** | 0.01 (-0.06-0.09) | **0.29 (0.19-0.38)** | Reference |  |
| Mean difference adjusted* ^a^ | **0.23 (0.03-0.44)** | 0.01 (-0.07-0.09) | **0.29 (0.19-0.38)** | Reference |  |
| Mean difference adjusted** ^a^ | **0.23 (0.03-0.44)** | 0.01 (-0.07-0.09) | **0.29 (0.19-0.38)** | Reference |  |
| SD- standard deviation, CI - confidence interval | |  |  |  |  |
| *Adjusted for age and gender (95% CI) |  |  |  |  |  |
| **Adjusted for age, gender and parental mental health (95% CI) | |  |  |  |  |
| ^a^ Mean difference in mean duration of visits from linear regression analysis | | |  |  |  |
| ^b^ Mean differences not estimated due to low cell count | |  |  |  |  |

**Table A2.** Mental and physical health of children and mental health of parents as predictors of health and school service use in the past 12 months among 3- to 5-year-olds (n =3,090)

|  | Type of Problems | | | |
| --- | --- | --- | --- | --- |
|  | Mental health and somatic problems | Mental health problems only | Somatic problems only | No problems |
|  | (n=21) | (n=173) | (n=108) | (n=1623) |
| Type of service | OR(CI) | OR(CI) | OR(CI) |  |
| *Model 1* |  |  |  |  |
| General Practitioner | **4.47 (1.51-19.14)** | 0.83 (0.60-1.14) | **4.57 (2.71-8.27)** | Reference group |
| Inpatient care | **9.37 (2.11-29.73)** | 1.68 (0.57-4.06) | **5.86 (2.64-12.04)** | Reference group |
| Extra help at school | **35.01 (13.31-90.43)** | **5.00 (2.62-9.17)** | **12.23 (6.74-21.87)** | Reference group |
| Extra help at home | **8.84 (2.81-23.51)** | **2.16 (1.08-3.99)** | **6.50 (3.66-11.18)** | Reference group |
| Any services | **3.69 (1.24-15.80)** | 1.06 (0.76-1.50) | **5.90 (3.21-12.17)** | Reference group |
|  |  |  |  |  |
| *Model 2* |  |  |  |  |
| General Practitioner | **4.41 (1.48-18.91)** | 0.82 (0.60-1.13) | **4.62 (2.73-8.37)** | Reference group |
| Inpatient care | **8.68 (1.95-27.73)** | 1.60 (0.54-3.88) | **5.72 (2.57-11.77)** | Reference group |
| Extra help at school | **33.58 (12.58-88.23)** | **4.78 (2.50-8.81)** | **12.00 (6.58-21.59)** | Reference group |
| Extra help at home | **9.15 (2.83-25.29)** | **2.02 (1.00-3.77)** | **6.78 (3.76-11.89)** | Reference group |
| Any services | **3.65 (1.23-15.65)** | 1.06 (0.75-1.49) | **5.92 (3.21-12.20)** | Reference group |
|  |  |  |  |  |
| Model 3 |  |  |  |  |
| General Practitioner | **4.39 (1.47-18.85)** | 0.82 (0.59-1.13) | **4.62 (2.73-8.37)** | Reference group |
| Inpatient care | **8.58 (1.92-27.54)** | 1.59 (0.53-3.86) | **5.72 (2.57-11-79)** | Reference group |
| Extra help at school | **32.14 (11.89-85.20)** | **4.25 (2.18-7.96)** | **12.36 (6.77-22.26)** | Reference group |
| Extra help at home | **9.40 (2.90-26.12)** | **2.08 (1.03-3.89)** | **6.75 (3.74-11.84)** | Reference group |
| Any services | **3.61 (81.21-15.48)** | 1.04 (0.74-1.48) | **5.92 (3.21-12.21)** | Reference group |
| OR - Odds ratio, CI - Confidence interval | | | |  |
| Model 1- logistic regression | |  |  |  |
| Model 2 - logistic regression controlling for child age and gender | | | |  |
| Model 3 - logistic regression controlling for child age, gender and parental mental health | | | | |

**Table A3.** Mean (SD) annual cost (USD 2016) of services used by children ages 3-5, by mental and somatic health status

|  |  | Type of Problems | | | | All children |
| --- | --- | --- | --- | --- | --- | --- |
|  |  | Mental health and somatic problems | Mental health problems only | Somatic problems only | No problems |  |
|  |  | (n= 21) | (n= 173) | (n= 108) | (n= 1623) | (n= 1925) |
| Type of service | | mean (sd) | mean (sd) | mean (sd) | mean (sd) | mean (sd) |
| **General Practitioner** | |  |  |  |  |  |
|  | Based on number of visits | 758.10 (836.32) | 235.81 (419.43) | 444.06 (482.57) | 209.05 (276.96) | 352.20 (321.67) |
| **Inpatient care** | |  |  |  |  |  |
|  | Based on number of days | 590.86 (2255.24) | 29.88 (173.73) | 296.80 (1462.64) | 26.12 (243.52) | 131.33 (1716.16) |
| **Extra help at school** | |  |  |  |  |  |
|  | Total cost extra help at school | 23362.86 (70278.60) | 849.25 (10096.07) | 19184.03 (61797.41) | 104.17 (1911.67) | 1848.34 (14846.94) |
| **Extra help at home** | |  |  |  |  |  |
|  | Total cost extra help at home | 2794.29 (12235.58) | 20.81 (127.05) | 716.25 (6321.41) | 24.18 (251.19) | 109.47 (1550.97) |
| **Total costs** | | 27506.1 (77782.93) | 1135.76 (10087.47) | 20641.14 (64625.06) | 363.52 (1977.47) | 2441.35 (15676.70) |
| SD - standard deviation | | |  |  |  |  |
